# Implications for paediatric training and workforce from pandemic disruptions: A view from a tertiary hospital

**DOI:** 10.1111/jpc.16180

**Published:** 2022-08-24

**Authors:** Jye Gard, Angela Cisternino, Clare Polley, Amy Gray

**Affiliations:** ^1^ The Department of Paediatrics, Melbourne Medical School University of Melbourne Melbourne Victoria Australia; ^2^ Murdoch Children's Research Institute, The Royal Children's Hospital Melbourne Victoria Australia; ^3^ The Education Hub The Royal Children's Hospital Melbourne Melbourne Victoria Australia

## Abstract

**Aim:**

To understand the lived experience of paediatric trainees in relation to their educational opportunities, workforce roles and the interplay between them, during pandemic disruptions.

**Methods:**

Twenty paediatric trainees working at Australian paediatric hospitals during the time of COVID‐19 restrictions were interviewed between July and November 2020. Based on a phenomenological approach, the interviews examined junior doctors' experiences in relation to medical education, adaptive education modes, learning opportunities and their workforce roles during the pandemic. Qualitative inductive thematic data analysis was used to develop a cohort narrative.

**Results:**

Four overarching themes were identified regarding trainee perceptions of the impact of COVID‐19 restrictions on learning opportunities, both positive and negative. These were: impaired rapport building, altered team role, altered care and education affordances versus access. Participants felt ill‐equipped to provide optimal clinical care during virtual and stifled in‐person consultations, detached from the multidisciplinary team, that changed work roles diminished their professional self‐worth, and that online learnings were advantageous if rostering afforded opportunities to engage with them.

**Conclusion:**

To equip paediatric trainees for the next steps in their careers, we suggest the following areas of focus: the use of new tools of rapport, smart investment in clinical moments, reconnection of multidisciplinary teams and learning, the support of online learning infrastructure with protected education time and roadmaps for learning, and teaching on how to triage information sources and alongside clinical visit types.

## What is already known on this topic

1. Dynamic COVID‐19 restrictions have altered both paediatric training and health service delivery.
2. Digital tools, blended learning and novel adaptive teaching modes have been used to try and mitigate learning gaps.
3. It is unclear what trainees have gained and lost through changes to education delivery and workforce roles, and how gains or losses need to be considered in future training and care.

## What this paper adds

1. This study sheds light on the nature, depth and breadth pandemic impacts on paediatric trainees' self‐perceived education, work and training needs.
2. Unmet training expectations left in the wake of the COVID‐19 include assessment and management of common and important ENT and respiratory presentations, management of the severely unwell, procedural skill mastery and ability to navigate professional conversations (breaking bad news, medical handover, discussions with multidisciplinary peers).
3. Educators must proactively invest in learning opportunities which recognise potential gaps and build connections through learning communities, upskilling trainees to overcome barriers caused by care at a distance, radical curriculum change to reflect both urgent and long‐term learning needs and empower trainees to uphold their full scope of practice to ensure they are ready for the next step in their careers.

Dynamic COVID‐19 restrictions have challenged paediatric training and care.^1^ As physical and social distancing restrictions separated clinicians from families, patients and peers, our modes of service and education delivery adapted to close the gap.^2^, ^3^ Paediatric educators worked to straddle these moving parts and adapt to the changed learning environment for training doctors.

Junior doctors are often transient in rotating roles and are accustomed to acclimatising to new workplace settings. The pandemic, however, has presented a challenge of unscaled proportions, with disruptions to work patterns, responsibilities and contexts, but also immediate and long‐term training.^4^ Formal examinations were postponed; clinical encounters were cancelled or restricted to only those deemed essential; and bedside teaching was stifled or defaulted to virtual learning. In response to these compound impacts, medical education responded with digital learning tools, blended classrooms and virtual resources to bridge learning gaps with variable success.^1^, ^2^, ^5^

Many paediatric educators hoped that the pandemic might foster skills in self‐directed learning, flexibility and resourcefulness, and a proficiency in virtual medicine.^1^ Yet, it is still uncertain what has been gained, changed and lost.

In this study, we aimed to understand paediatric trainee experiences in educational opportunities, workforce roles and their interplay in the context of the COVID‐19 pandemic in Australia. In doing so, we hoped to think about how to optimise the education–workforce interface now and as we move through the pandemic.

## Methods

We conducted a qualitative study on in‐depth individual interview data, drawing on a phenomenological^6^ approach to understand a lived experience. This was to emphasise the personal perspective and interpretation of events by junior doctors and avoid assumptions about what topics might be important in the data. Junior doctors employed by a tertiary paediatric training hospital were invited to participate using the hospital email distributions lists. The hospital employs paediatric doctors training in a variety of subspecialties both within the hospital and at peripheral sites. All sites accept both emergency presentations from the community and elective admissions. At the time of the study, these health sites saw minimal coronavirus cases, large reductions in patient presentations (both overall and of specific illnesses) and changed work roles to maximise staff working from home and limit team exposure to possible infection. Health services anticipated an impending surge of presentations of critically unwell patients as seen overseas. Hurdle examinations for junior doctors to progress in their training were suspended and the potential extent of career disruption was yet unknown. At a health service level, the possibility of redeployment of staff to other services remained an active discussion and infection control precautions required staff to physically distance and prioritise who saw which patient.

Twenty training doctors; five from each key level of training (specialist training year 1 (STY 1), STY2, STY 3–4 and STY 5+) were identified via an opt‐in process to enable representation from different levels of seniority and therefore, potentially different experience. We monitored our data throughout for thematic saturation. Sixteen participants identified as female, four male. Interviews took place from July to November 2020.

Individual semi‐structured interviews were conducted via video link using open‐ended questions (Table 1). Interviews were audio‐recorded, transcribed verbatim, de‐identified, and then the audio‐recording was deleted. Written consent was obtained before interview, and participants were provided with a written copy of their de‐identified transcribed interview and the opportunity to withdraw their data in whole or in part within 2 weeks. There were no withdrawals.

**Table 1:** Interview guide

Questions	Prompts
Can you tell me about how the COVID‐19 restrictions impacted on your education, or education opportunities at work?	
Can you tell me about your experience of working and the changes which occurred in the work environment during COVID‐19?	What did you miss?
	What opportunities were created?
	What was difficult?
What education did you expect you would receive this year?	What was difficult?
How did this differ from what you have received and what new resources have you been using for learning?	What opportunities were created, or did you find?
What did you miss? Including any learning opportunities, you realised the value of when they were gone	
What activities or people/groups provided professional and/or educational support during COVID‐19?	
Can you tell us about the types of educational activities which might work best in the traditional face‐to‐face format?	
What opportunities have worked well in different contactless formats (e.g. not traditional face‐to‐face)	
Is there anything else you think is important to highlight about training during the COVID‐19 restrictions?	

Interviews were coded independently by two investigators following an iterative process with comparison. Coding was conducted using NVivo Release 1.4 (QSR International). Narrative analysis was then performed on the coded data through the lens of Bruner's functional approach.^7^ The study was approved by Human Research Ethics Committee (HREC Reference number: 64940).

## Results

We identified four central themes regarding paediatric practices and training changes due to COVID‐19 restrictions: impaired rapport building, altered team role, altered cares and education opportunities versus access (Table 2).

**Table 2:** Themes and participant quotes

Theme	Subtheme	Quote
Rapport toolkit	In‐person encounters	‘At one point … [hospitals] only allowed one parent in the emergency department. I think a lot of parents found it difficult. And that was … [a] lot of transference of emotion … like it was almost like you felt guilty because of what was being imposed on them. You couldn't really support them’. (Participant 3, ST4)
		‘We wear like full PPE for every single patient encounter and it feels so impersonal and so much scarier for the children … just that thing of being able to talk to a family or a child without PPE ….’ (Participant 5, ST2)
	Virtual encounters	‘Now we have a lot of outpatient work and a lot of that has become telehealth outpatient work. A whole part of paed[iatric]s learning is how to interact with the child and you know, getting the joy of interacting with the child as well … we don't actually examine the child and we take a lot of things on face value from the referral about whether they have hepatosplenomegaly or not’. (Participant 11, ST5+)
		‘We can do telehealth … but it's been the clinical skills, bonding with the family, getting like verbal cues and communications … [clinicians using telehealth] could not like really read the parents’. (Participant 15, ST4)
	Peer modelling and direct supervision	‘I got to spend less time with patients. And I wasn't allowed to be as inclusive – bring in my residents for all the interactions and things like that, because we're obviously trying to do everything kind of quickly, in and out …’. (Participant 9, ST3)
		‘So for our clinics at the moment … before the consultant would usually be in the clinic with you … whereas now they are usually upstairs in the office doing telehealth … and it is also great, excellent in fact, but just a different dynamic’. (Participant 16, ST5+)
	Impacts on professional identity	‘To me most of the things I've been missing are around [direct] patient interaction. I'm only in the hospital once every three weeks and then there's two weeks of very intermittent patient interaction that is almost all through Telehealth. So I do miss that’ (Participant 2, ST3)
		‘I feel like it's the reason I went in medicine … you don't even really get to have much of that chitchat that you're used to because you're always so conscious of your space’ (Participant 1. ST1)
Altered team role	Lost learnings	‘The emergency department didn't want inpatient teams coming down to examine the children they referred. So trying to make sensible plans over the phone from what was often the registrar's or resident's report who weren't paediatrically trained was hard’. (Participant 3, ST3)
		‘I really wasn't exposed to a child who was very unwell’. (Participant 2, ST2)
	Diminished responsibilities	‘… they're [the consultants] are on EPIC [an electronic medical record system] more … instead of being able to present a patient to a boss, you can't because they've already looked it all up on a computer. I think less input from us I guess, less opportunities to present and things’. (Participant 17, ST2)
		‘there was a registrar who was rostered to being “extra” on the ward, which meant that they were there to do procedures and those types of things where you don't need to have knowledge of the patient to support the team and everyone on. But since coronavirus restrictions have started, they are reducing the total number of people on ward, which makes sense, they've kept a fellow on the ward to do all the procedures because they can do them without any assistance and then us registrars are in‐office doing paperwork like discharge summaries and things’. (Participant 11, ST5+)
	Clinical team disruption	‘… there was a real lack of those informal and formal social opportunities with colleagues. And the same with things like sitting in the meeting room with the multidisciplinary team and actually knowing who people are rather than just a name or a video on the screen’. (Participant 17, ST1)
		‘I definitely value how we used to have a cast of thousands … social workers, nurses …’ (Participant 18, ST2)
	Reduced consultant contact	‘There's been less of “I'll just come and watch you” from a learning perspective … it's more about “Do I really need to be there or can you just go and do it and then we'll manage and talk about it after kind of from a far’. (Participant 4, ST3)
	Virtual barriers	‘But now with Zoom because as a registrar you just feel more like you're down the rung and less able to offer your opinions. I don't know it just feels more awkward for some reason. (Participant 18)’ (Participant 6, ST4)
		‘I think as you go through training, the purpose … is to get you ready for when you're a consultant down the road. I think decreased exposure to senior clinicians affects that in lots of ways … your ability to learn from your bosses … in normal times, [this] happens a lot through chit‐chat at the end of ward rounds. Your social relationships you know build into professional ones that get you to where you want to go’. (Participant 2, ST1)
		‘I think a lot of medical practice especially in junior years is very comradery based and I think a lot of people use that as a way to survive or even to have a way to what you're really doing at the end of the day … the current restrictions have really impeded a lot of that’. (Participant 1, ST1)
		‘Sometimes you feel a bit like you don't know quite where you sit when there's all these things are going on and all the decisions are being made at a much higher level’. (Participant 6, ST3)
Altered care	Limited examination	‘Think about things that you would normally do as part of the work up you'd do you know you would do your cardio and ENT when you first meet kids especially when I was on overnight ‐like if children were undergoing aerosol generating procedure, ED would do it, you wouldn't do it, and you'd just take their word for it’. (Participant 3, ST2)
		‘[Previously] you would actually see the child face to face and examine them properly and then you sort of order investigations based off that but now you sort of go “oh yeah” well someone wrote that they had that, so we'll just do all of this’. (Participant 12, ST3)
	Information gathering	‘I was actually finding that workflow was quite challenging, because there might have been five referrals that I would normally be able to go down and admit then hand over to the single registrar on overnight … it created a whole lot of (extra) work …’ (Participant 14, ST3)
	Consultation delays	‘And actually being able to link into the subsequent specialties and things like that – everything has just become a little bit more time intensive and a little bit trickier to facilitate and keep on top of just that administration … I guess because no one is physically there in person and you're not able to just have that in the moment conversation’. (Participant 18, ST1)
	Stifled assessments	‘And a lot … has become telehealth outpatient work. Often I don't even see the child as part of the parent interaction …’ (Participant 15, ST2)
		‘I guess sometimes missing the examination can make your assessment trickier or it just means you end up doubling up on appointments because you see a patient via telehealth and you realise, “Oh, we actually should have seen them in person.” So then they're coming in anyway’. (Participant 19, ST4)
		‘The assessment of developmental stuff I think currently that that's really hard to do, it used to be that kids would look at you and something that you do and be like “ooh”’. (Participant 17, ST4)
Educational affordances versus access	Mixed benefits	‘Because we didn't have to go to where it was being held face to face, it was almost a little bit easier in that respect, because you could sign on from anywhere’ (Participant 4, ST1)
		‘I signed up to a conferences last week that is now being held online that I would have most certainly not been able to … it's just more accessible and I'm more likely to go to that. And then again that flexibility of being able to listen to some sessions on the day but listen to a majority later on’. (Participant 3, ST2)
		‘It's [the roster] been good because I can keep up to date … I can use [the time afforded] to keep up with international colleagues and see if they have any nice infographics or teaching resources … I've also started using the textbooks that I previously bought … I'm listening to podcasts more’. (Participant 13, ST4)
		‘I think if anything I feel like I'm kind of Zooming into these different things while the kids are running around my feet, or I'm listening to a podcast while I'm cleaning – it just feels like there more multi‐tasking this year than there ever has been for me. I would love to sit down and read a textbook but that's not the reality for me at the moment’. (Participant 15, ST5+)
	COVID‐19 dominance	‘I feel that every week [my seniors were] talking about new protocols and having a new staff screening or new things we have to do, or be aware of, like PPE … it's all consuming’ (Participant 2, ST1)
		‘I feel like a lot of the time was spent talking about COVID rather than taking [up] learning opportunities’ (Participant 1, ST2)

### Impaired rapport building

All participants reported that wearing personal protective equipment (PPE) significantly hindered their ability to build rapport with children and their families. Distraction and engagement tools were removed for infection control purposes. Junior doctors perceived this detrimentally impacted the quality of care and communication they provided. Most also felt ill‐equipped to establish rapport during non‐face‐to‐face encounters, and this further hindered their ability to contextualise, navigate and provide reassurance during complex discussions. Moreover, many participants felt they were not taught how to tailor their approach to overcome these barriers to rapport building.

With limited numbers of clinicians at the bedside, trainee doctors from all levels of seniority, opined that they were the first ones to be excluded. Many lamented the lost opportunity to observe and model senior clinicians' approach to clinical encounters. Others explained that this diminished rapport with peers and patients, confidence in clinical abilities, perceived value within the clinical team, agency to build a professional identity, and job satisfaction.

### Altered team role

Participants perceived a loss of opportunities to learn specific clinical examinations, communication, disease management and technical subspecialty skills (Table 3). Most recognised that this was in part due to reduced hospital presentations, but also asserted that this was compounded by shielding – restricted access to areas of the hospital, cares, and types of patients. The processes and approaches that influenced these experiences were decided locally.

**Table 3:** Perceived losses in education and training opportunities

Domain	Learnings
Examination skills	Examinations that increase the risk of droplet/aerosol infection
	Approaches to the paediatric examination
	Building family rapport
	Examining children in ED and other high‐risk settings
Communication	Presenting patients
	Breaking bad news
	Participating in multidisciplinary meetings
Altered patient cohort	Respiratory conditions (e.g. bronchiolitis and use of high‐flow support)
	Increased mental health and injury cases
	Severely unwell
Procedural tasks	Bedside procedures (e.g. cannulation and lumbar puncture)
	Assisting in surgery
	Interpreting imaging

Participants reported that the scope of their responsibilities changed and that many former roles were adopted by senior team members. These ranged from team communication and clerking patients to clerical duties. Missed bedside opportunities were not well met by other education modes such as online clinical meetings.

All participants felt estranged from peers, clinical teams and patients. Interestingly, many highlighted being especially distanced from their interdisciplinary and allied health colleagues with whom they only interacted virtually. Many participants felt disempowered in virtual meetings and were reluctant to contribute without opportunity to first build rapport with the faces on screen. Most also highlighted the palpable absence of the at‐elbow consultant to provide direct supervision, expertise and feedback. Most felt that the level of supervision and support previously provided in‐person, diminished when provided at a distance. This impacted participants' perceived value and contribution to the multidisciplinary team. Without rapport and connection with teams, many felt a lack of self‐agency; voiceless, unclear of their professional identity and unable to envisage their future career.

### Altered care

To limit direct patient interaction and the risk of COVID‐19 spread, participants tailored their physical examinations and did not repeat examinations previously documented and relied on these for decision‐making. Furthermore, many placed greater onus on colleagues' opinions, workups and were expected to make clinical decisions remotely.

As many outpatient encounters were triaged as virtual visits to mitigate infection risk, junior doctors had to carefully consider the risks and benefits of conceding components of the physical examination unable to be attended online, the accuracy of those recorded by colleagues and the value of second in‐person consultation. If it was difficult to engage children via video or phone, many participants shifted to engaging the parent alone. Virtual and physically distanced visits restricted the performance and gathering of information from specific assessments, such as developmental examinations.

### Educational opportunities versus access

During the study period, many hospital meetings, conferences and junior doctor‐specific teaching sessions moved online. Subspecialty departments and colleges worked to translate educational content online (e.g. bedside tutorials to videos). Participants readily articulated the benefits afforded by the new modes of education delivery including improved access to content, no commutes, unrestricted attendance numbers and greater freedoms around time. They felt this occurred at the expense of reduced context for learning, direct supervision, nuance in skill development and mastery of high‐level communication skills.

Access to online education opportunities were highly influenced by workforce roles (e.g. enhanced by shift‐based work with rostered education time), workloads as well as home stressors. Participants who were not afforded protected time, perceived online and other asynchronous education opportunities as ‘homework’; learnings to be engaged with outside of work hours. Most felt that there was an unspoken expectation to engage with any new online content made available to them and some felt a sense of ‘failure’ if they did not.

Participants perceived that COVID‐19 learnings (e.g. PPE training, infection control techniques and public health policy discussions) dominated the education narrative. Furthermore, many felt these learnings, although needed, ‘got in the way’ of learning content more important for their future practice.

## Discussion

Taken together, our findings show that the pandemic hindered our paediatric trainees' ability to work, learn and connect. Our participants felt ill‐equipped to provide optimal clinical care during virtual and stifled in‐person patient encounters, detached from the multidisciplinary team, and unsure of how to achieve the learning targets expected of them. Changed work roles and responsibilities diminished our junior doctor's sense of professional self‐worth, functions within the clinical team and often saw them entrust aspects of cares they would usually provide, to others. The ability to engage with online and asynchronous learning opportunities was heavily influenced by how their workforce role was structured. Our study identified potential key targets for paediatrics and its workforce going forward: to address learning gaps in how to provide best care at a distance, undo pandemic ways of working, optimise workforce roles to support education access in all modalities (face‐to‐face, online and self‐directed), and ensure clinical education continues to support clinical team cohesion and connection.

As direct patient interaction was limited and modified, many junior doctors felt disempowered to approach the bedside and escalate care when they found the restricted encounter insufficient. Although trainees were able to tailor components of their clinical encounter in the name of infection mitigation and efficiency, many also ‘made do’ with the information made available to them. It is important to ensure necessity does not become habit, and solutions may require us to re‐emphasise clinical reasoning based on accurate data gathered from patients.^8^ Education should also focus on empowering the junior doctor to articulate and enact needed steps in care – including those we used to take for granted, such as seeing patients face‐to‐face. Supervising clinicians need to be aware of potential practices which may become engrained and explicitly enquire about the source of information under‐pinning judgements or gaps in experience.

Accustomed to directly supervised practice and the traditional doctor apprenticeship model,^9^ many of our participants struggled with working remotely and distanced teamwork. Classically, one of the integral roles of a junior doctor is that of the medical team and patient liaison.^10^ As this and other responsibilities were increasingly shouldered by other members of the medical team, many of our junior doctors perceived their personal value and contribution to clinical care were diminished. Furthermore, many cited that the absent at‐elbow consultant made it difficult for them to find their place in the team and training trajectory. Mentorship remains a key pillar for junior doctor development^9^ and investment in connections is needed after pandemic disruptions. Time should be invested in empowering trainees to take a more significant role in shaping their own professional identity in times of disruption and to understand the impact afterwards; what has been gained or lost.

The ways we learn and teach have expanded over the last 2 years, yet the structure of workforce roles and hospital education programs have not necessarily changed to think about how we support flexibility in trainee learning. Set education times may not be achievable or accessible due to work demands, but allocated time for self‐directed learning using the wealth of resources available is also not embedded in organisations. To capitalise on the changing shape of education, workplace agreements must leverage flexibility in education requirements. As the benefits of online and asynchronous learning are harnessed, the significant role clinical education provides in fostering professional connections and context for training, must not be forgotten.^11^, ^12^ Although the virtual world can serve some functions, face‐to‐face opportunities are critical to help junior doctors find their place and professional identify in teams.

A major learning gap in our participants' training was rapport building skills. In a paediatric context, the therapeutic relationship is paramount in promoting patient and, especially in young children, family engagement in all aspects of care.^13^, ^14^ Our findings highlighted that many strategies our junior doctors use to build family rapport rely on visual stimuli, such as distraction, modelling and mirroring. Mask‐wearing specifically has been found to increase the difficulty in engaging with young children^15^, ^16^ and their in‐hospital use is likely to be sustained.^17^ Other techniques should be explicitly taught to maximise patient engagement and minimise fear,^18^ such as dynamic storytelling, humour and vivid imagery.^19^ In addition, the role of aerosol protecting illustrated or clear masks should be considered.^17^ As digital health technologies continue to evolve, it is important to examine when and how virtual care is best tailored to patient needs and incorporate this in trainee education. Moreover, it is vital that future studies examine the virtual visit ‘tipping point’ – the contexts in which the use of new digital tools begins to degrade the therapeutic relationship.

The strength of this study is to give a voice to junior doctors from a range of levels of seniority, and that data saturation was reached during the interview process. However, the study represents one hospital, at one time, during a pandemic which had varying and dynamic local and global consequences, so it cannot be representative of all junior doctor experiences. There may be selection bias in that in those who volunteered for the study in terms of their interest in education and their desire to report their experience, with potential exclusion of those who felt disconnected from the hospital learning community, burnt out or disillusioned.

## Conclusion

Our junior doctors described a cognitive dissonance between work‐based and online resources. Many felt the changes to their workplace, roles and responsibilities were transient and thus learnings gained in this context were not to be prized. By contrast, most of the education offered online taught the ‘traditional curriculum’; clinical presentations and care that were commonly seen prior to the COVID‐19 pandemic. The result was that many junior doctors felt that the workplace was for service delivery only and that ‘true’” education occurred online and often after hours when not supported by their rosters or roles.

As we emerge from the pandemic, care must be taken to understand its lasting footprint on junior doctor education and workplace roles, both positive and negative. As a rotating, and therefore transient workgroup in a disrupted hospital system junior doctors deserve specific attention.

To equip paediatric trainees for the next steps in their careers, we suggest the following areas of focus: the use of new tools of rapport, smart investment in clinical moments, reconnection of multidisciplinary teams and learning, the support of online learning infrastructure with protected education time and roadmaps for learning, and curriculum changes aimed to teach how to triage information sources and alongside clinical visit types. Junior doctors should be intimately involved in the design and delivery of new curriculum or programs. Harnessing their unique insights into the contexts surrounding teaching and achieving new learning objectives is vital for success.
